# Photo-Magnetic Irradiation-Mediated Multimodal Therapy of Neuroblastoma Cells Using a Cluster of Multifunctional Nanostructures

**DOI:** 10.3390/nano8100774

**Published:** 2018-09-29

**Authors:** Rohini Atluri, Rahul Atmaramani, Gamage Tharaka, Thomas McCallister, Jian Peng, David Diercks, Somesree GhoshMitra, Santaneel Ghosh

**Affiliations:** 1 Nano-Bio Engineering Laboratory, Southeast Missouri State University, Cape Girardeau, MO 63701, USA; atluri.rohini@gmail.com (R.A.); rratmaramani1s@semo.edu (R.A.); tgmccallister@gmail.com (T.M.); somesree@gmail.com (S.G.); 2 Mechanical and Energy Engineering Department, University of North Texas, Denton, TX 76207, USA; 3 Department of Bioengineering, The University of Texas at Dallas, Richardson, TX 75080, USA; 4 Department of Physics and Engineering Physics, Southeast Missouri State University, Cape Girardeau, MO 63701, USA; tharakarcbb13@gmail.com (G.T.); jpeng@semo.edu (J.P.); 5 Department of Metallurgical and Materials Engineering, Colorado School of Mines, Golden, CO 80401, USA; ddiercks@mines.edu

**Keywords:** photo-magnetic actuation, cisplatin, nanoparticle, MYCN, multimodal therapy

## Abstract

The use of high intensity chemo-radiotherapies has demonstrated only modest improvement in the treatment of high-risk neuroblastomas. Moreover, undesirable drug specific and radiation therapy-incurred side effects enhance the risk of developing into a second cancer at a later stage. In this study, a safer and alternative multimodal therapeutic strategy involving simultaneous optical and oscillating (AC, Alternating Current) magnetic field stimulation of a multifunctional nanocarrier system has successfully been implemented to guide neuroblastoma cell destruction. This novel technique permitted the use of low-intensity photo-magnetic irradiation and reduced the required nanoparticle dose level. The combination of released cisplatin from the nanodrug reservoirs and photo-magnetic coupled hyperthermia mediated cytotoxicity led to the complete ablation of the B35 neuroblastoma cells in culture. Our study suggests that smart nanostructure-based photo-magnetic hybrid irradiation is a viable approach to remotely guide neuroblastoma cell destruction, which may be adopted in clinical management post modification to treat aggressive cancers.

## 1. Introduction

Neuroblastoma is a childhood cancer that is diagnosed at a median age of 17 months [1], with an incidence rate of 10.2 per million children under 15 years of age [2]. There are about seven hundred new cases each year in the United States, and in two out of three cases, the disease usually spreads to the lymph nodes or other parts of the body at the time of diagnosis. This is an embryonal tumor of the autonomic nervous systems [3], and it is the most common extra cranial tumor of childhood with long term survival rates of only about 15% [4]. Theoretically, tumors can appear anywhere along the sympathetic nervous system, but in reality, a majority of the tumors are detected in the adrenal medulla [5]. Other sites for tumors include the upper chest, neck, and paraspinal spaces. Often, metastasis can be seen in regional lymph nodes and in the bone marrow, and during an advanced stage of the disease, it can infiltrate a local organ such as with a celiac axis tumor [6,7]. Overexpression and dominance of cell survival pathways are mainly responsible for the malignant transformation and metastasis of neural crest derived cells [8]. There are several factors that define specific cases of neuroblastoma, but high risk ones include stage, age, MYCN oncogene amplification, chromosome 11q status, metastasis, histologic category, and deoxyribonucleic acid (DNA) ploidy [5]. 

Due to biological heterogeneity of neuroblast tumors, different therapeutic strategies are pursued. While reduced intensity therapeutic approaches, such as surgery alone or in combination with moderate intensity chemotherapy, are the usual line of treatment for less aggressive tumors, high intensity chemo-radiotherapies are usually favored for tumors with more aggressive features [5]. For high risk neuroblastoma, the current treatment is divided into three phases: (1) induction of remission, (2) consolidation of remission, and (3) maintenance. The most commonly used induction regimen includes cycles of cisplatin and etoposide as well as alternate use of vincristine, doxorubicin, and cyclophosphamide [9]. Additionally, two types of radiation therapies are used: (1) external beam radiation therapy, and (2) Metaiodobenzylguanidine (MIBG) radiotherapy [10]. Myeloablative chemotherapy with autologous hematopoietic stem-cell rescue [4,11] and isotretinoin with anti-GD2 immunotherapy [12] is also considered for high-risk neuroblastoma treatment. 

Although the use of high-intensity chemo-radiotherapies have demonstrated only modest improvement in the treatment of high-risk neuroblastoma, undesirable side effects include mouth sores, nausea, hair loss, and most importantly, increased chance of infection [10]. In addition to these, there may be several drug-specific side effects, for example, cisplatin and carboplatin can affect kidneys [13], doxorubicin is a cardiotoxic agent [14], and cyclophosphamide can damage bladder as well as ovaries and testicles [15], which may affect future fertility. Short-term side effects of radiation therapy are nausea, diarrhea, burns, and fatigue [10], while long-term side effects may lead to damage in DNA, which has a risk of developing into a second cancer many years after completion of radiotherapy. Unfortunately, despite implementing all advanced treatment modalities, 50–60% patients in high-risk groups have a relapse, and there is no known curative treatment available to date [5]. Use of anti-GD2 monoclonal antibodies to prevent relapse is a good example of an immunotherapeutic approach to lessen the side effects of chemo [16], as well as radiotherapies. A future trend is to develop antibody-based treatment guidelines as well as synergistic combination therapies. 

From the above discussion, it is evident that innovative approaches possessing a novel therapeutic potential need to be implemented in order to overcome the existing challenges to treat high-risk neuroblastoma. An innovative technique that holds promise in the area of cancer diagnosis and therapeutics to perform precise drug delivery, multimodal therapy, and detection of circulating or residual cancer cells, all of which can play crucial roles in the treatment of high-risk neuroblastoma, is the development of novel nanostructures coupled with smart actuation strategies [17,18,19,20,21,22,23,24]. Nanostructured materials and smart surfaces carry excellent treatment potential for the development of novel clinical solutions because they can be designed to target/detect specific cancer cells and be remotely tuned to release measured doses of therapeutic agents, which in turn may improve treatment efficacy, decrease therapy time, and decrease the quantities of the therapeutic agent necessary for effective treatment by 10–50-fold [25]. In order to meet these goals cumulatively, “combinatorial therapeutics” approaches consisting of various nanostructures and advanced instrumentation are becoming one of the most exciting forefront fields, but it has been in its infancy until now. Oscillating magnetic field induced hyperthermia [26,27,28] or photothermal destruction of cancer cells [29] are among the most promising approaches among these; however, both fall short of addressing several concerns, including the use of high intensity magnetic or optical irradiation coupled with a lower yield at a clinically viable dose level. As discussed earlier, the rapid emergence of treatment resistance is a formidable challenge that needs a multimodal treatment approach, and unfortunately, the aforementioned approaches do not address this concern. Recently reported [30] “multimodal chemo-radiotherapy of glioblastoma” demonstrated encouraging outcomes, which has the potential of addressing this challenge; however, the technique needs further investigation before successful implementation, especially where the use of potent γ–rays is involved. 

Therefore, we set ourselves the goal of enhancing the treatment efficacy by combining a group of smart nanostructures, each of which are capable of performing a specific task with a novel strategy that has been unexplored thus far, namely simultaneous photo-magnetic actuation. In this study, three different types of nanostructures have been used to accomplish the objectives: (1) core-shell magnetic nanospheres (CSMNSs), (2) Polyvinylpyrollidone (PVP)-capped gold nanoparticles (AuNPs), and (3) cisplatin loaded thermo-responsive nanoparticles (CPNPs). The first two protagonists (i.e., the CSMNS and the AuNPs) induce a coupled hyperthermia and oxidative stress under the hybrid photo-magnetic irradiation, whereas the CPNPs cause sustained release of the imbibed cisplatin during the treatment. These augmented the cisplatin and photo-magnetic hyperthermia mediated cytotoxicity inducing mechanisms, and intensified the oxidative stress induced damage, all at a relatively lower irradiation and nanoparticle exposure level, which led to complete ablation of the B35 neuroblastoma cells in culture. Additionally, by using this technique, exposures to the high energy γ-rays have been avoided. Our study suggests that smart nanostructure-based photo-magnetic hybrid irradiation is a viable approach to remotely guide neuroblastoma cell destruction, which may be adopted as an efficient technique in clinical management post modification. Although we have explored this technique for neuroblastoma cell destruction in this study, it can be further modified and extended to treat other aggressive cancers.

## 2. Materials and Methods 

### 2.1. Photo-Magnetic Actuator Design

**Figure 1:**
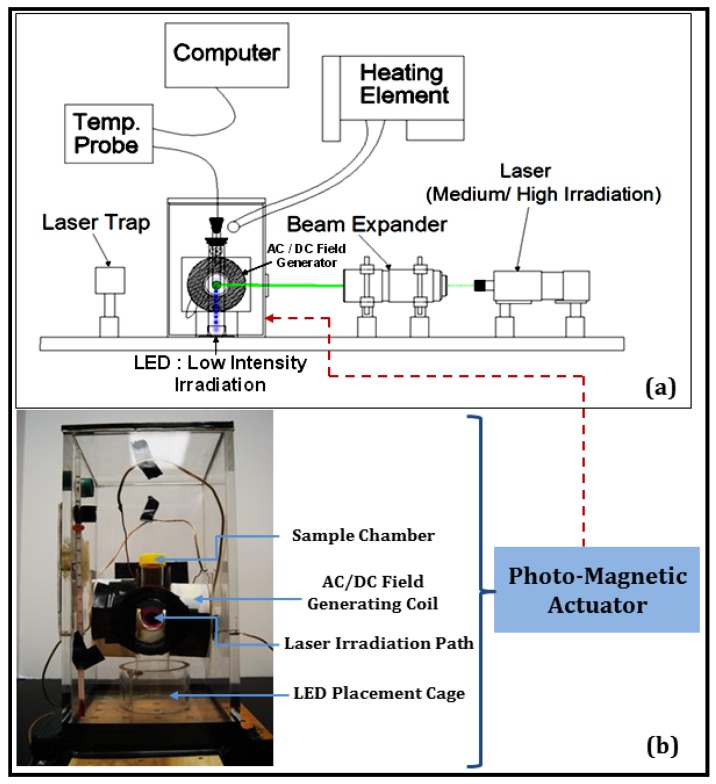
(**a**) Schematic of the experimental setup for the combined optical-AC magnetic field irradiation of nanocarriers and B35 neuroblastoma cells (electronics not shown). (**b**) Various components of the incubator with a TPP tissue culture tube mounted inside.

A unique photo-magnetic actuator was designed to perform simultaneous optical and AC magnetic field stimulation of cultured mammalian cells or dispersed nanocarrier systems (Figure 1a,b). The incubator (Figure 1b) consisted of a sample chamber for placing TPP tissue culture tubes, AC/DC magnetic field generating coil, a cage for the placement of light-emitting diodes (LEDs) for low-level optical irradiation, and a high-performance glass window at the front wall of the incubator for transmitting the laser irradiation during moderate/high level optical stimulation. Inside the sample chamber, the B35 neuroblastoma cells were cultured or the nanocarriers were colloidally dispersed, as needed. The circuit utilized a capacitor bank in series with the inductor coil and a 0.5 Ω resistor. A magnetic field in the range of 10–150 Oe and 60–150 kHz could be produced as needed by changing the capacitor and/or the coil inductance. A temperature controlling unit was attached to stabilize the incubator temperature in the range of 36–37 °C during the experiments, and the top and the bottom panels of the incubator were designed to be removable to allow easy swapping of the samples. The incubator was attached to the base of the class 3B laser (520 nm, 300 mW), and a laser stop was placed to the rear of the incubator to inhibit reflection. Further, black absorbent tape material was used to confine the laser exposure to only necessary areas. A fiber optic thermometer was used to measure precise temperature change during heating of the nanocarriers.

### 2.2. Nanocarrier Design 

Magnetite (Fe_3_O_4_) core-polymeric shell nanospheres (CSMNS) were synthesized as reported in our previous work [31]. In brief, a double-layered shell consisting of a thermo-activated polymer network of poly(ethylene glycol) ethyl ether methacrylate-*co*-poly(ethylene glycol) methyl ether methacrylate (PEGEEMA-*co*-PEGMEMA) was synthesized first using a precipitation polymerization method. One batch of these designed spheres was used to induct the magnetic nanocrystals inside the outer shell, while the other batch was freeze dried and later loaded with the anticancer drug cisplatin (Sigma Aldrich, Bellefonte, PA, USA), as described below. Polyvinylpyrollidone (PVP)-capped gold nanoparticles (0.05 mg/mL, 5 nm diameter) were obtained from nano Composix. All nanocarrier morphology was assessed by performing scanning and transmission electron microscopy (SEM and TEM: FEI NOVA 230 NANOSEM, Tustin, CA, USA, accelerating voltage 5–20 kV; Philips EM 420 TEM, Port Elizabeth, South Africa-120 kV electron beam) [31].

### 2.3. Loading the Drugs in Polymeric Nanocarriers and Characterization of Release Profile

An aqueous solution of cisplatin (2 mg/mL) was added to the previously prepared freeze-dried (non-magnetic) nanospheres. The solution was stirred for 24 h at room temperature and the cisplatin-loaded nanoparticles (CPNPs) were collected by centrifugation. A specialized diffusion chamber (PermeGear Static Franz cell,) with two compartments was used for the in vitro release kinetics measurement. The two compartments communicate through an opening 2 cm in diameter. A semipermeable membrane (mol. wt. cut-off 13,000 Da) was used to cover the opening. The CPNP solution was placed in the donor compartment and the receiver compartment was filled with the deionized (DI) water. To determine the concentration of the released cisplatin (at room temperature and at 37 °C) in the receiving compartment, samples were withdrawn at definite time intervals (20, 40, 60, 80, and 100 h) and the absorbance was measured using UV-visible spectroscopy at a wavelength of 301 nm.

### 2.4. Light Scattering and Magnetic Measurements

Dynamic light scattering (DLS) measurements were performed to examine the volumetric transition behavior using a Malvern NanoZS system equipped with a helium-neon laser (632.8 nm) as the light source. The hydrodynamic radius distribution of the nanospheres in water was measured at a scattering angle of 60°. A magnetic property *[M(H)]* of the nanospheres was measured using a Lakeshore model 7300 (Westerville, Ohio, USA) Vibrating Sample Magnetometer (VSM), at ambient temperature and at 38 °C. 

### 2.5. Cell Culture and Treatment

B 35 rat neuroblastoma cells (ATCC, Manassas, VA, USA) were routinely cultured at 37 °C in 5% CO_2_ and 85% relative humidity by using Dulbecco’s modified Eagle’s medium (DMEM, Invitrogen, Carlsbad, CA, USA) derived complete media that contains 90% DMEM, and 10% fetal bovine serum (FBS). For the experiments, about 10,000 cells/cm^2^ were seeded in TPP tissue culture tube flasks (10 cm^2^ growth surface area) containing 2 mL of DMEM complete media and were allowed to grow for 48 h or more until a 70% confluence was observed. All the experiments were performed in triplicates. 

For the treatment with nanoparticles, after 48 h of cell growth and attachment, the cells were washed with serum-free DMEM and were exposed to the NPs (various concentrations of MNPs (Magnetic Nano-Particle) and/or AuNPs), which were colloidally suspended in the culture media. During the nanoparticle exposure, cultures were placed into serum-free DMEM to prevent particle aggregation. After 4 h of exposure, the cells were washed with serum-free DMEM and were cultured back into 2 mL complete DMEM media until the beginning of the next exposure cycle. The treatment was repeated thrice for every 24 h. At the end of the final exposure, live cell imaging was performed to assess the cell proliferation.

For the AC magnetic field exposure, optical irradiation, and hybrid optical-AC magnetic field exposure, the cells were cultured and exposed to the NPs as mentioned earlier. Immediately after the addition of NPs (MNPs and/or AuNPs), the cells were exposed to AC magnetic field exposure/optical irradiation/hybrid optical-AC magnetic field exposure (magnetic field intensity 60 Oe, frequency 120 kHz, laser power 300 mW) for 15 min. Following irradiation, the cells were placed in the incubator for 3 h and 45 min as part of the treatment. After 4 h of NP exposure and irradiation, the cells were washed with serum-free DMEM and were cultured back into 2 mL complete DMEM media until the beginning of the next exposure cycle. The treatment was repeated thrice for every 24 h. At the end of the final exposure, live cell imaging was performed to assess cell proliferation.

For the treatment with cisplatin-loaded thermo-responsive nanoparticles (CPNPs), after 48 h of cell growth and attachment, the cells were washed with serum-free DMEM and were exposed to the CPNPs (200 µg/mL), which were colloidally suspended in the culture media for 4 h. After 4 h of exposure, the cells were washed with serum-free DMEM and were cultured back into 2 mL complete DMEM media until the beginning of the next exposure cycle. The treatment was repeated thrice for every 24 h. At the end of the final exposure, live cell imaging was performed to assess cell proliferation. 

For hybrid optical-AC magnetic field exposure in the presence of CPNPs and NPs, the cells were treated (with CPNPs and NPs) as mentioned earlier. After the addition of the nanocarriers, the cells were exposed to hybrid optical-AC magnetic field exposure (magnetic field intensity 60 Oe, frequency 120 kHz, laser power 300 mW) for 15 min. Following irradiation, the cells were placed in the incubator for 3 h and 45 min as part of the treatment. After 4 h of nanocarrier exposure and irradiation, the cells were washed with serum-free DMEM and were cultured back into 2 mL complete DMEM media until the beginning of the next exposure cycle. The treatment was repeated thrice for every 24 h. At the end of the final exposure, live cell imaging was performed to assess cell proliferation.

Nuclear morphology was assessed using confocal images captured through a 64× objective from cells (cultured on the coverglasses, which were inserted into the TPP tissue culture tube flasks and fixed) labeled with 4′,6-diamidino-2-phenylindole (DAPI, Ex = 405 nm, Em = 450/35 nm), following various treatments.

### 2.6. Flow Cytometry Analysis: Annexin V Apoptosis Assay

Upon treatment under various conditions, the cells were washed with serum-free DMEM, trypsinized, centrifuged, and suspended in 500 µL 1× binding buffer. Cells were further incubated with FITC (Fluorescein isothiocyanate) Annexin V apoptosis detection reagent for 20 min at room temperature in darkness (100 µL of cell suspension was mixed with 5 µL of FITC Annexin V and 5 µL of PI), followed by flow cytometry analysis.

## 3. Results

A simultaneous optical and AC magnetic field assisted therapeutic strategy was unexplored thus far, despite having a huge potential of generating synergetic effects, which may be especially beneficial for the destruction of aggressive cancer cells. This innovative setup (Figure 1a,b) enabled high-risk neuroblastoma cell exposure to varying combinations of optical and magnetic field excitation in the presence of specifically designed nanocarriers, thereby augmenting the positive outcomes of separate actuation strategies and the nanocarrier functionalities. The maximum field strength generated by the coils (≈150 Oe) is approximately 200 times weaker than that produced by a magnetic resonance imaging (MRI) machine (≈3 × 10^4^ Oe), which are known to be safe for use by people with medical implants such as pacemakers [32]. It may be noted that a Helmholtz coil-based design can be adopted for conducting experimentation with animal models and to obtain deeper penetration, a near infrared (NIR) laser can be used [22]. However, for low-level photo-magnetic therapy requiring LED irradiation in vivo, further modification is needed in the instrumentation.

**Figure 2:**
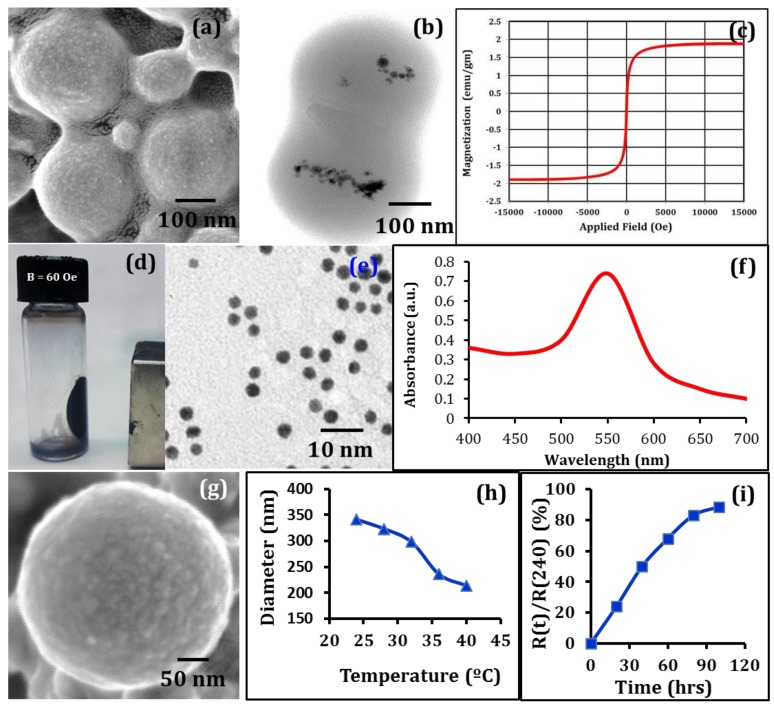
Morphology of designed CSMNSs: (**a**) SEM analysis demonstrating the polymer shell, and (**b**) TEM analysis demonstrating the distribution of the encapsulated MNPs. (**c**) Applied field vs magnetization plot for CSMNSs at 311 K, demonstrating super-paramagnetic behavior, even at the collapsed state of the polymeric shell. (**d**) Response of the CSMNSs to an applied DC magnetic field of 60 Oe by a permanent magnet at the adjacent wall of the flask. Characterization of the AuNPs: (**e**) TEM analysis demonstrating the particle distribution in cell culture media, ruling out the possibility of agglomeration, and (**f**) UV-visible spectrum of the dispersed AuNPs in the culture media. (**g**) SEM imaging of the CPNP demonstrating nanocarrier morphology. (**h**) Temperature dependence of hydrodynamic diameter of the CPNPs. (**i**) Drug release profile from the CPNPs. R(t) represents the mass released at any time t, and R(240) represents total mass released over 240 h (10 days).

No recognizable physicochemical interactions or clustering (and thereby precipitation) were observed among these three types of nanocarriers when they were (simultaneously) dispersed for 48 h in: (i) aqueous solution (DI H_2_O), and in (ii) phosphate buffered saline (PBS). This indicates that the encapsulation (shell) formed by the polymerized, stable, and higher mechanical strength-possessing PEG-derivative biopolymer chains protected the embedded magnetic nanoparticles from being exposed to the proteins, salts, and other potential reactive agents present in the colloidal suspensions. Similarly, the polyvinylpyrollidone surface-tethered gold-nanoparticles were protected from potential (damaging) interactions with the media constituents, and therefore, did not facilitate agglomeration. The synthesized magnetic nanocarriers (CSMNSs) exhibited good colloidal stability, strong magnetic properties, and no precipitation after several days. From the SEM imaging, slightly oval shaped particles (arising from the surface roughness of the carbon film during sample preparation), were observed (Figure 2a). The mean diameter of the nanocarriers was found to be 268 ± 24 nm. Particle encapsulation was assessed using TEM imaging at 120 kV. The resulting TEM micrographs (Figure 2b) revealed that the magnetic nanocrystals were located near each other, which is very typical for magnetic nanoparticle-based systems, as observed earlier by several researchers [26,33,34]. Due to their size and structure, the nanomagnets were expected to exhibit super-paramagnetic behavior at a moderate field and frequency (0–150 Oe, 0–1000 kHz) range [31,35], which was assessed at 311 K, or above the volumetric transition temperature (Figure 2c). No to minimal hysteresis response was observed, unlike the ferromagnetic nanoparticle-based systems [28,34], even after the volumetric shrinkage of the spheres, which indicated super-paramagnetic behavior and the absence of nanocrystal agglomeration at elevated temperatures. Under the measured field intensity of 60 Oe, created by a permanent magnet at the adjacent wall of the flask, the CSMNSs moved in the direction of the field and formed a film on the flask wall close to the magnet (Figure 2d). Almost all particles were completely separated from the solution, even with the application of a moderately intense field, which demonstrated their controllability under a magnetic field. It may be noted that the CSMNS response to an external magnetic field was much stronger than that of the individual magnetic nanodots due to a much higher magnetization value per carrier. Slight agitation brought the nanospheres back into the solution once the magnetic field was removed. This behavior further indicated that it will be possible to trap and maintain these nanocarriers in the targeted tissue regions without being washed away by the blood flow during in vivo applications. TEM imaging of the AuNPs demonstrated the particle distribution (Figure 2e) in the culture media and the high absorption in the range of 520 ± 15 nm (Figure 2f) facilitated coupled hyperthermia under hybrid optical-AC magnetic field exposure, as assessed later. The CPNPs consisted of two polymer shells with varying degrees of hydrophilicity, as described in the previous section (nanocarrier design), the inner one having a diameter of 162 ± 24 nm (not shown here). Multi-shell nanocarriers were designed to expand the volumetric transition range [31], which in turn facilitated the release of the imbibed therapeutic agents. Morphology of the CPNPs was assessed by performing SEM imaging (Figure 2g) and the mean diameter of the double shell nanocarriers was found to be 341 ± 32 nm. The temperature-dependent volumetric transition behavior of these cisplatin loaded nanocarriers is shown in Figure 2h, which demonstrates a broader (31–38 °C) volumetric transition range, and consequently, sustained release of the imbibed cisplatin (Figure 2i). 

**Figure 3:**
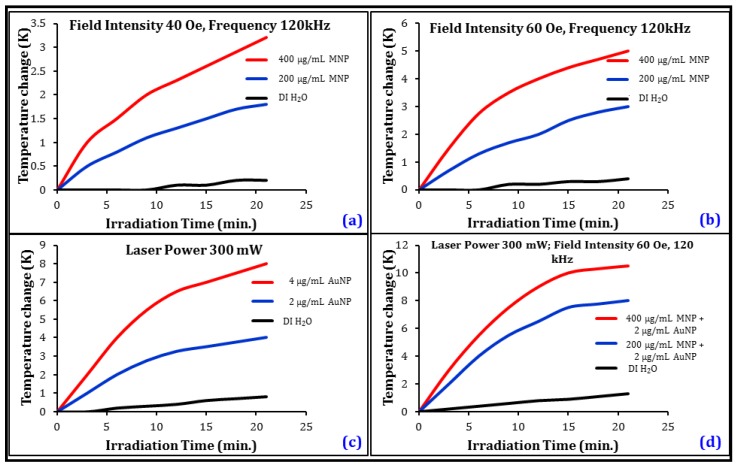
Remote heating response under AC magnetic field exposure as a function of CSMNS concentration at (**a**) 40 Oe, and (**b**) at 60 Oe. Frequency of the magnetic field was kept at 120 kHz. Heating response (**c**) under optical irradiation as a function of AuNP concentration, and (**d**) under hybrid optical-AC magnetic field irradiation using CSMNSs and AuNPs together in the media at various concentrations.

The remote heating response of the nanocarriers was observed under AC magnetic fields (Figure 3a,b), optical irradiation (Figure 3c), and under hybrid optical-AC magnetic field exposure (Figure 3d). Upon field application, the nanocarrier-suspended culture media temperature increased in a concentration-dependent manner and reached a near steady state after approximately 20–30 min of irradiation. For AC magnetic field modulation, MNP concentration was varied between 200–400 µg/mL, and the temperature change was observed to be in the range of 1.5–3.5 K at 40 Oe, and between 3–5 K at 60 Oe field intensities, respectively. The optical irradiation-induced temperature change was found to be in the range of 3.7 and 8 K, respectively, when the concentration of the AuNPs were changed from 2 to 4 µg/mL in the culture media. A significantly stronger heating response was observed under the hybrid optical-AC magnetic field, in the range of 8–10.5 K, even with a mixture consisting of only 2 µg/mL AuNPs and 400 µg/mL MNPs. During all measurements, observed joule heating was found to be minimal, in the range of 0.5–1.25 K. Observing the heating response under coupled optical-AC magnetic field and considering clinically viable dose levels of the nanocarriers, 400 µg/mL MNPs and 2 µg/mL AuNPs were chosen as the mixture composition for executing acute hyperthermia towards the development of a multi modal therapy for the destruction of the neuroblastoma cells.

**Figure 4:**
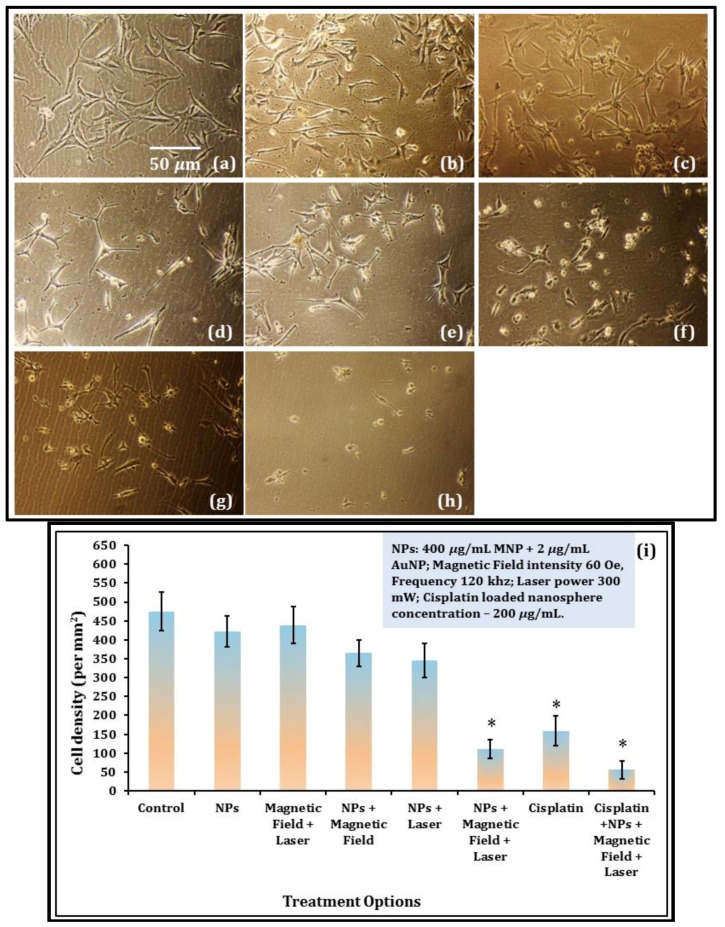
B35 neuroblastoma cell proliferation under the following conditions: (**a**) control; (**b**) presence of NPs (400 µg/mL MNP + 2 µg/mL AuNP) in the culture media; (**c**) combined optical-AC magnetic field irradiation in the absence of NPs; (**d**) presence of NPs under AC magnetic field irradiation; (**e**) presence of NPs under optical irradiation; (**f**) combined optical-AC magnetic field irradiation in the presence of NPs; (**g**) presence of 200 µg/mL CPNPs; and (**h**) combined optical-AC magnetic field irradiation in the presence of NPs and CPNPS. The AC magnetic field intensity was 60 Oe, frequency was 120 kHz, and laser power was 300 mW (at 520 nm). Scale bar is 50 µm in (**a**), and is also applicable for (**b**–**h**). (**i**) Bar chart displaying quantification of average cell densities (cell number/mm^2^), indicative of cell proliferation for B35 neuroblastoma cells under all treatment options. Data are means ± SEM from four separate experiments and * indicates statistically significant differences, compared to cells cultured as the control, at *p* < 0.01 (ANOVA and LSD post-hoc).

B35 neuroblastoma cell proliferation was observed post hyperthermia treatments and compared with the control (Figure 4a), and with only nanoparticle exposure (Figure 4b) conditions. The dose level of the CSMNSs and AuNPs used in this study were found to have a very low cytotoxicity, as observed in Figure 4b and quantified later (Figure 4i). Hybrid optical and AC magnetic field irradiation did not inhibit cell proliferation in the absence of the nanocarriers, as observed in Figure 4c, although a slight reduction in cell proliferation was observed in the presence of the nanocarriers under separate (i.e., magnetic or optical) actuations (Figure 4d,e). Under combined photo-magnetic actuation in the presence of the nanocarriers, severe inhibition in proliferation with cytoplasmic blebbing and irregularities in shape were observed (Figure 4f), which even surpassed the culture condition with the CPNP exposure in the media (Figure 4g). Finally, complete ablation of the B35 neuroblastoma cells in culture was observed under photo-magnetic combined actuation in the presence of the magnetic, gold, and the cisplatin loaded nanocarriers (Figure 4h). One-way analysis of variance (ANOVA) depicted statistically significant differences (*p*-value < 0.01) in the cell density values between the control and hybrid photo-magnetic actuation in the presence of nanocarriers with and without the presence of the cisplatin loading, as well as between control and 200 µg/mL CPNP treatments (Figure 4i).

**Figure 5:**
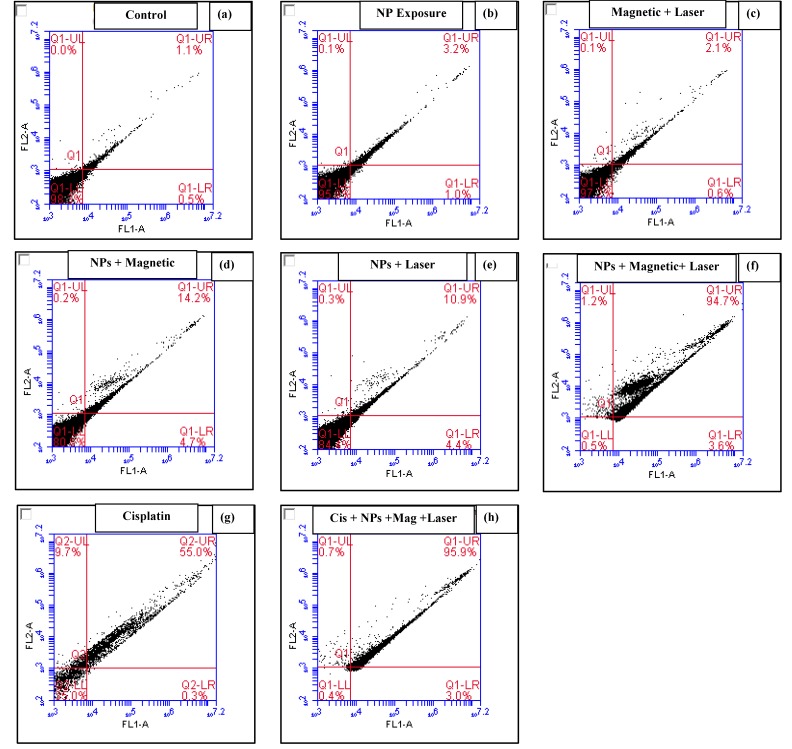
Apoptosis detection by the Annexin V assay. In four windows of each plot, the lower left indicates normal cells, the lower right indicates early apoptotic cells, the upper right indicates middle phase apoptotic cells, and the upper left indicates late phase apoptotic cells or necrotic cells. Irradiation/exposure parameters where applied: AC magnetic field intensity was 60 Oe, frequency was 120 kHz, and laser power was 300 mW (at 520 nm); NPs: (400 µg/mL MNP + 2 µg/mL AuNP); CPNPs 200 µg/mL.

In apoptotic cells, the membrane phospholipid phosphatidylserine (PS) is translocated from the inner to the outer leaflet of the plasma membrane, thereby exposing PS to the external cellular environment. Annexin V is a 35–36 kDa Ca^2+^ dependent phospholipid-binding protein (conjugated to FITC) that has a high affinity for PS, and binds to cells with exposed PS. Staining with FITC Annexin V is typically used in conjunction with a vital dye, such as propidium iodide (PI) or 7-amino-actinomycin (7-AAD), to identify early apoptotic cells. Cells that are considered viable are FITC Annexin V and PI negative; cells that are in early apoptosis are FITC Annexin V positive and PI negative; and cells that are in late apoptosis or already dead are both FITC Annexin V and PI positive. Results demonstrated more than 95% viable cells for the control (Figure 5a), post nanoparticle exposure (Figure 5b), and hybrid photo-magnetic irradiation in the absence of the nanocarriers (Figure 5c), thereby revealing the innate biocompatibility of the nanocarriers, as well as the irradiation exposure. Slight elevation of apoptosis (15–20% in the suspended cells) was observed in the presence of the nanocarriers under separate (i.e., magnetic or optical) actuations (Figure 5d,e). However, 98% early/late apoptotic cells were observed under combined photo-magnetic actuation in the presence of the nanocarriers (Figure 5f), which was found to be significantly greater than that of the 200 µg/mL CPNP exposure (65% apoptotic/necrotic cells, Figure 5g), demonstrating the extent of induced cytotoxicity by this hybrid actuation-nanocarrier combination. Figure 5h demonstrates a severe degree of induced apoptosis (99% apoptotic or necrotic cells) under photo-magnetic combined actuation in the presence of the magnetic, gold, and the cisplatin loaded nanocarriers. It should be noted that the apoptosis trend was found to be somewhat similar to the previously observed cell proliferation results.

**Figure 6:**
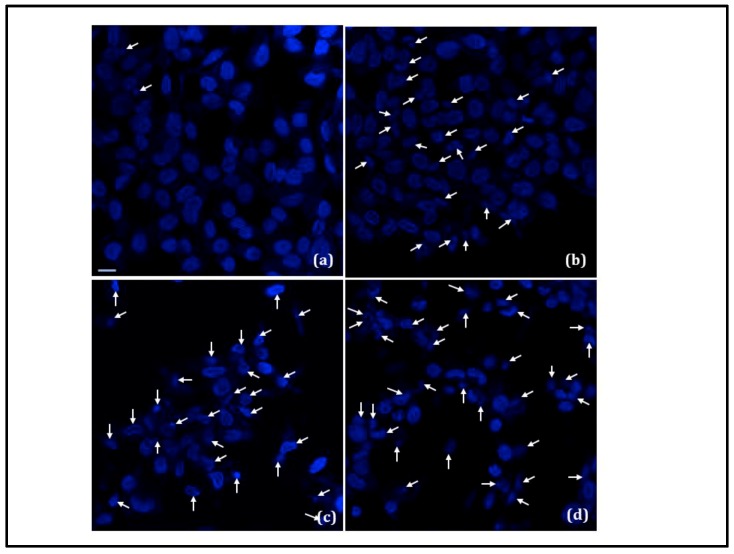

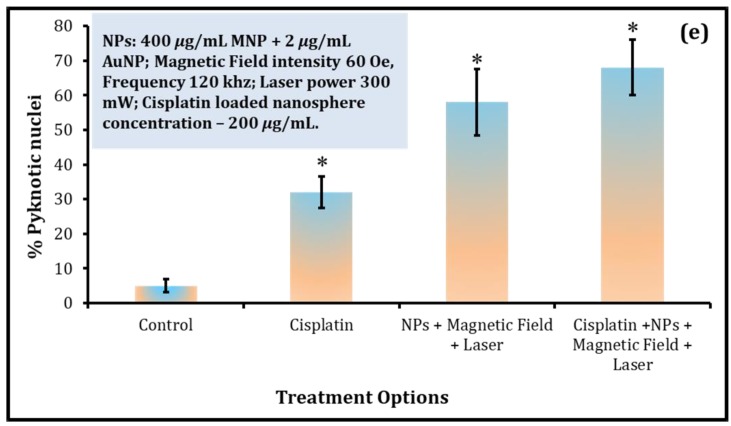
Nuclear condensation and fragmentation (white arrows) under the following conditions: (**a**) control, (**b**) presence of 200 µg/mL CPNPs, (**c**) combined optical-AC magnetic field irradiation in the presence of NPs (400 µg/mL MNP + 2 µg/mL AuNP), and (**d**) combined optical-AC magnetic field irradiation in the presence of NPs and CPNPS. AC Magnetic field intensity was 60 Oe, frequency was 120 kHz, and laser power was 300 mW (at 520 nm). Scale bar is 10 µm in (**a**), and is also applicable for (**b**–**d**). (**e**) Bar chart displaying quantification of pyknotic nuclei, indicative of cell death. Data are means ± SEM from four separate experiments and * indicates statistically significant differences, compared to cells cultured as the control, at *p* < 0.01 (ANOVA and LSD post-hoc).

Nuclear changes, such as condensation of the nucleus and/or DNA fragmentation, are the typical characteristics of later stages of the apoptotic program. Induction of apoptosis was further investigated by observing DAPI stained cell nuclei for the conditions that severely inhibited B35 neuroblastoma cell proliferation (Figure 6a–d). While in the control (Figure 6a), the cells had round and homogeneous nuclei, exposure to CPNPs (200 µg/mL) launched the apoptotic machinery of the cell, as observed from the deformed and condensed nuclei and apoptotic bodies (Figure 6b). Under combined photo-magnetic actuation in the presence of the gold and magnetic nanocarriers, severe chromatin condensation and nuclear fragmentation was evident (Figure 6c), indicating the potency of photo-magnetic hyperthermia-mediated cytotoxicity at a relatively lower irradiation and nanoparticle exposure level. Even a higher degree of damage was observed under photo-magnetic combined actuation in the presence of the CSMNSs, AuNPs, and CPNPs (Figure 6d), thereby demonstrating the effectiveness of the multimodal therapeutic strategy. Quantification of pyknotic nuclei, which is indicative of cell death [36], is displayed in Figure 6e, depicting statistically significant differences (*p*-value < 0.01) between the control and 200 µg/mL CPNP treatment, as well as between the control and hybrid photo-magnetic actuation in the presence of nanocarriers with and without the presence of the cisplatin loading.

## 4. Discussion

**Figure 7:**
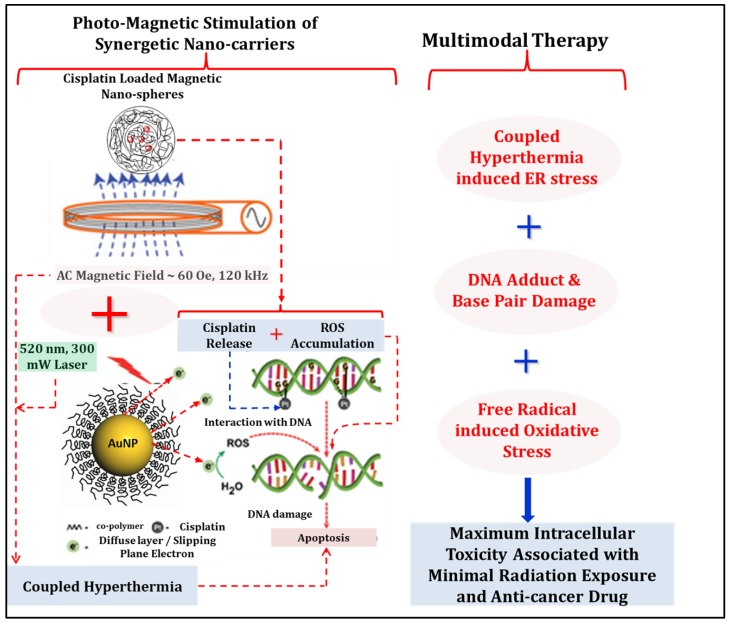
Photo-magnetic irradiation mediated multimodal therapeutic strategy of neuroblastoma cells using clusters of nanostructures: coupled hyperthermia, DNA damage, and reactive oxygen species (ROS) -induced apoptosis of B35 neuroblastoma cells in culture. Note that in this experiment, cisplatin has been loaded in a separate non-magnetic nanocarrier.

Combined photo-magnetic stimulation has successfully been implemented on the cluster of complementing nanocarriers to develop a multimodal therapy to guide the neuroblastoma cell destruction (Figure 7). This novel strategy permitted the use of a less intense AC magnetic field in combination with optical irradiation during the treatment, thus removing the safety concerns associated with the AC magnetic field-assisted therapies. Although a green laser (300 mW) has been used in this study as the light source for the optical irradiation as a proof of concept, it can be replaced by a near infrared (NIR) laser to obtain deeper penetration, since the gold nanoparticles can be tuned to possess high NIR absorption [22]. The penetration depth of the optical irradiation can be further enhanced by the use of free-space or even a fiber-optic Bessel beam [37], thus eliminating the use of high-intensity radiotherapy, which has the potential to incur severe DNA damage and has a risk of developing into a second cancer at a later stage. Moreover, the treatment efficacy has been achieved at a reduced nanoparticle dose level [28,38]. In our recent reports [21,24], various strategies for targeting and delivery of therapeutic agents for the central nervous system (CNS)-related conditions have been identified: (i) endocytosis based, and (ii) laminin (or other disease specific surface proteins) binding peptide based. The later strategy is gaining huge traction for specific targeting at present, and coupled with the impressive development of the target-specific synthetic oligonucleotides/aptamer design [39], provides a viable option for delivery of these nanocarriers, since all these vectors can be surface-functionalized with appropriate functional groups (such as –COOH, –NH_2_, or –SH) for the conjugation of biomolecules. Another recent work by Jeong et al. [40] demonstrated the feasibility of administering these types of nanocarriers intravenously to treat spinal cord injury in mice. Prior observations indicate that these aforementioned strategies will increase the concentration of the nanocarriers at the target tissue. Site-specific injection is also another route that needs to be explored with these types of nanocarriers depending on the location and accessibility of the tumor. In our previous studies [24,31,41], the nanocarriers were found to be highly non-reactive, stable in physiological solutions, and were minimally toxic at even a higher dose level than the dose administered here. Reduced dose level can potentially render them as ideal candidates for photo-magnetic combination therapy.

For tumorigenesis and malignant transformation, responsible molecular mechanisms are: (1) overexpression of cell survival pathways, and (2) downregulation of apoptosis [8]. The molecular factors of cell survival pathways include protein kinases (protein kinase B (AKT/PKB); anaplastic lymphoma kinase (ALK); phosphatidylinositide 3-kinases (PI3K); and focal adhesion kinase (FAK)), transcription factors (NF-κB, MYCN, and p53), and growth factors (insulin-like growth factor (IGF); epidermal growth factor (EGF); platelet-derived growth factor (PDGF); and vascular endothelial growth factor (VEGF)). Manipulation of the cell survival pathways may reduce the malignant potential of the tumor, which in turn may provide reduction of required dosages and the dose-related side effects of the conventional therapies in clinical practice. Moreover, since the presence of residual cancer cells in the hematopoietic compartment is the plausible explanation for tumor relapse [5], highly sensitive methods to detect and isolate rare circulating tumor cells may lead to improved treatment efficacy. The cluster of nanostructures used in this study carries the potential to act as effective modulators of these pathways and selectively target the tumor cells due to their controllability under hybrid photo-magnetic field and temperature-sensitive behavior. A temperature-dependent hydrophilic–hydrophobic transition behavior renders them suitable for drug delivery applications as well, in which triggered release is necessary.

Kinases are enzymes to phosphorylate, thus they act as on-off switches for activating other factors in cell signaling pathways. One well known kinase is AKT kinase, which regulates important cellular functions like cell growth, proliferation, survival, and angiogenesis [8,42]. In human tissue samples, it was observed that the AKT phosphorylation was more prevalent in primary neuroblastoma than in benign ganglioma or in normal adrenal tissue [8]. Downregulation of AKT to increase apoptosis is one of the many ways to address the neuroblastoma tumor growth, and two main strategies are being pursued: (1) long-term exposure of SH-SY5Y cells to interferon β, which decreased activation of the P13K-AKT pathway [43,44], thereby increasing the apoptosis, and (2) Rapamycin-induced mTOR (a downstream effector of AKT) inhibition [45], which is related to decreased tumor growth, angiogenesis, and increased apoptosis. Similarly, inhibition of FAK by siRNA [46] or small molecule inhibitors, such as NVP-TAE 226 [47] and Y15 [48] results in decreased cell survival, increased apoptosis, and G2 cell cycle arrest. NVP-TAE 226 (mol. wt. 468.94) and Y15 (mol. wt. 284.01) are ideal candidates to be loaded into these designed nanocarriers due to their low molecular weight and adequate water solubility, which will be extremely beneficial for controlled release into the tumor cells under photo-magnetic stimulation. Among transcription factors, NF-κB has important roles in neuroblastoma chemo-resistance as doxorubicin and VP16 have both been shown to trigger NF-κB activation in neuroblastoma cells, inhibiting apoptosis [49]; nevertheless, siMYCN (siRNA against MYCN) has been found to increase caspase-3 mediated apoptosis [50]. Selective inhibition of MYCN can be achieved using an anti-gene peptide nucleic acid (PNA) [51], which can either be covalently attached to the nanocarrier surface, or can be loaded inside for on-demand release when the target site is reached. Targeted therapy to modulate the growth factors is another direction for the treatment of high risk neuroblastoma [52]. Imatinib, a tyrosine kinase inhibitor of PDGFR (PDGF receptor) has been shown to inhibit the growth of a number of human neuroblastoma cell lines in vitro and xenograft in vivo [53]. We recently demonstrated a nanocarrier mediated neurite growth factor (NGF) delivery to neuronal model cells for promoting neurite outgrowth [28,41]. A similar strategy can be adopted for the delivery of a selected growth factor mediated cell survival pathway modulators to the targeted cancer cells. 

For high-risk neuroblastoma treatment, identification and targeting of the rare circulating tumor cells or removal of the nucleic acids from such cells is extremely important to prevent the tumor relapse. We have recently designed Förster resonance energy transfer (FRET)-based multifunctional nanocarriers [41], which are capable of performing organelle specific binding for detection of damaged cells and can provide on-demand release of a specific drug or a combination of drugs. Combined with the photo-magnetic actuation, these nanocarriers have the potential to perform detection at the single cell level, which may lead to a greater understanding of how to handle residual tumor cells. Further, since most of these aforementioned tasks can be performed with various types of magnetically controllable nanocarriers, it will be possible to prevent the diffusion out of the targeted area using a concentrated DC magnetic field during in vivo localization. Use of a Halbach cylinder [54] can extend the penetration depth of the applied magnetic field during clinical applications.

## 5. Conclusions

In conclusion, an optical and AC magnetic field-assisted therapeutic strategy for high risk neuroblastoma treatment was developed. Multifunctional nanostructures CSMNSs, AuNPs, and CPNPs at a reduced dose level were used to create coupled hyperthermia and induce sustained release of the imbibed cisplatin, which caused complete ablation of the B35 neuroblastoma cells. This enabled replacement of high energy γ–ray and high-intensity AC magnetic field exposure. The developed technique can potentially further combine the modulation of cell survival pathways and the detection of rare circulating tumor cells, thereby leading to a greater understanding and comprehensive solution to overcome the existing challenges to treat high-risk neuroblastoma. The results of this study suggest that photo-magnetic irradiation based multimodal therapy is a viable approach to remotely guide neuroblastoma cell destruction and the technique may be extended to treat other aggressive cancers.
